# A study of target effect sizes in randomised controlled trials published in the *Health Technology Assessment* journal

**DOI:** 10.1186/s13063-018-2886-y

**Published:** 2018-10-10

**Authors:** Joanne C. Rothwell, Steven A. Julious, Cindy L. Cooper

**Affiliations:** 1 School of Health and Related Research, University of Sheffield, Sheffield, UK; 2 Sheffield Clinical Trials Unit, University of Sheffield, Sheffield, UK; 3 The Medical Statistics Group, School of Health and Related Research (ScHARR), University of Sheffield, Regent Court, 30 Regent Street, Sheffield, S1 4DA UK

**Keywords:** Randomised controlled trial, Target difference, Effect size, HTA, Health technology assessment

## Abstract

**Background:**

When designing a randomised controlled trial (RCT), an important consideration is the sample size required. This is calculated from several components; one of which is the target difference. This study aims to review the currently reported methods of elicitation of the target difference as well as to quantify the target differences used in Health Technology Assessment (HTA)-funded trials.

**Methods:**

Trials were identified from the National Institute of Health Research *Health Technology Assessment* journal. A total of 177 RCTs published between 2006 and 2016 were assessed for eligibility. Eligibility was established by the design of the trial and the quality of data available. The trial designs were parallel-group, superiority RCTs with a continuous primary endpoint. Data were extracted and the standardised anticipated and observed effect size estimates were calculated. Exclusion criteria was based on trials not providing enough detail in the sample size calculation and results, and trials not being of parallel-group, superiority design.

**Results:**

A total of 107 RCTs were included in the study from 102 reports. The most commonly reported method for effect size derivation was a review of evidence and use of previous research (52.3%). This was common across all clinical areas. The median standardised target effect size was 0.30 (interquartile range: 0.20–0.38), with the median standardised observed effect size 0.11 (IQR 0.05–0.29). The maximum anticipated and observed effect sizes were 0.76 and 1.18, respectively. Only two trials had anticipated target values above 0.60.

**Conclusion:**

The most commonly reported method of elicitation of the target effect size is previous published research. The average target effect size was 0.3.

A clear distinction between the target difference and the minimum clinically important difference is recommended when designing a trial. Transparent explanation of target difference elicitation is advised, with multiple methods including a review of evidence and opinion-seeking advised as the more optimal methods for effect size quantification.

## Background

The major funder of research into clinical interventions in the United Kingdom (UK) is the National Institute of Health Research (NIHR), and the biggest programme within that is the Health Technology Assessment Programme (HTA). The HTA funds commissioned and researcher-led health-related research including randomised controlled trials (RCTs) of clinical interventions in the UK [1, 2].

One of the conditions of funding from the HTA is that all studies must write a HTA report to be published in the *Healthy Technology Assessment* (*HTA*) journal. Many trials which are funded by the HTA are also published in journals such as the *Lancet*, the *British Medical Journal* and the *New England Journal of Medicine*. However, the HTA publishes all reports for trials it funds, irrespective of the statistical significance achieved, and these reports have greater detail than journal articles can include. Therefore, journals published in the *HTA* journal are suitable for review as they are published in detail, are of high scientific standard and are published regardless of the positive or negative nature of the results.

A key component when designing a clinical trial is the sample size justification. If there are too few participants then the trial may not result in statistical significance even if there is a true effect [3]. Conversely, having too many participants could result in unethical practice; for example, randomising unnecessary numbers of participants to a treatment which could may be shown to be inferior or harmful earlier and delaying the results of the study [3].

The most sensitive part of the traditional sample size calculation is the anticipated difference or effect size between treatments. This difference can be categorised as either a clinically meaningful difference or a target difference. A clinically meaningful difference is the value above which you would accept that one treatment is clinically superior to another. However, it may not always be desirable to use a clinically meaningful difference. It could be that we need to demonstrate a difference greater than the minimum clinically meaningful difference to influence medical practice or policy. The target difference may then be set higher than the minimum clinically meaningful difference. Throughout this paper we will use target difference when talking about the effect size.

The elicitation of this target difference is a widely discussed issue, with a large review being performed in 2014 by Cook et al. which showed that a variety of methods are used in establishing a target effect size [4, 5]. This study draws from the findings of the DELTA project, a Medical Research Council (MRC)-funded study which resulted in the publication by Cook et al., and has been performed as part of the DELTA2 project, also funded by the Medical Research Council (MRC). The purpose of the DELTA2 project is to formulate guidance on choosing the target difference for RCTs, aiming to assist trialists in the design of trials. This study uses the definitions of target difference elicitation methods developed by the original DELTA project in the review.

This study aims to assess the currently reported methods of elicitation of the target difference as well as quantify the target differences used in HTA-funded trials.

## Methods

### Trial identification

A review of RCTs published in the *HTA* journal between 2006 and 2016 was performed. This time frame was chosen primarily because based on an initial scoping study to assess if there were sufficient eligible reports, as well as being recent and manageable for the author in the time frame. The use of the *HTA* journal as the data source for this study means that both statistically significant and non-significant trials are included, since the journal reports trials irrespective of their resulting statistical significance. This ensures that reporting bias is not thought to be an important problem in this study. Without the implications of reporting bias, and the high level of detail that is included in *HTA* journal reports, the choice of the *HTA* journal allows greater understanding and transparency.

The search criteria consisted of including only RCTs with a parallel-group design which had the objective to assess superiority. The reason for this decision was due to the parallel-group design being the most commonly undertaken. This was confirmed by an initial scoping of the HTA report.

The scoping consisted of assessing volumes 19 and 18 for the number of reported RCTs and their designs. The proportion of reports which were concerned with RCTs in these volumes were 23.9 and 20.6% for volumes 18 and 19, respectively. Of these RCTs, the percentage of parallel-group superiority RCTs was 78% for volume 19 and 80% for volume 18.

Further exclusions were trials which did not contain the enough information for appropriate analyses to be performed, trials with more than three arms due to the additional complexities involved in co-primary endpoints and vaccination trials which also had multiple primary endpoints. These multiple primary endpoints resulted in more than one target difference in the various sample size calculations, making data extraction complex.

### Data extraction

Each trial included had a unique identifier the International Standard Randomised Controlled Trial Number (ISRCTN). Data that could not be extracted from the included trials were denoted as ‘Missing’.

Data extraction was completed using a series of Microsoft Excel spreadsheets with a large variety of variables and free-text boxes for further information if required. A full list of extracted variables can be seen in the Appendix. The extraction was carried out by one reviewer over a period of 9 months. All categorical variables were coded prior to completion of data extraction, with further additions to the coding if this provided clarity for various design features. For example, the clinical areas and elicitation methods were amended during data extraction to provide more information, as described in the next section.

### Categorisation of variables

In the event of a categorical variable being subjective in nature, or outside the immediate understanding of the reviewer, further advice was sought. This occurred for two variables, the clinical area of the trial and the target effect size elicitation method.

For the clinical categorisation, data were initially categorised into 15 clinical areas. At an interim assessment point, however, a large number of trials fell into the ‘Other’ category (18.7%). Advice provided by a physician resulted in a further five clinical categories which were Renal/Urology, Special Senses (Ear, Nose and Throat (ENT) and Ophthalmology), Geriatrics, Critical Care, Emergency Care and Lifestyle. After extraction, categories which were only assigned to one trial were combined into an ‘Other’ category to reduce the large number of categories. The combined categories were Haematology, Emergency Care and Primary Care.

The category labelling (or describing) the target difference elicitation methods was handled in a different manner. This was based on that used by Cook [4]. This used seven broad categories which are the methods of:

- Anchor
- Distribution
- Health economic
- Opinion-seeking
- Pilot study
- Review of evidence-base method
- Standardised effect size

These methods are described briefly, with further information found in a publication by Cook et al. [4, 5].

#### Anchor method

This method starts by establishing the anchor, by calculating a mean change in ‘score’ for patients who have expressed that a minimum clinically important difference or change has occurred in the context of quality-of-life measures [6, 7]. This change in their quality of life measure can then be evaluated and used as a clinically important difference in future trials using the same outcome measure. It then tries to implement the minimum clinically important difference (MCID) found in the first part. This will change depending on the measure being used.

Another variation of this method is to ‘anchor’ a new outcome measure to a previously used outcome measure, when both measures are correlated [8, 9]. An example of this would be trying to implement a new quality of life (QoL) measure or subscale, and anchoring it to a generic QoL questionnaire.

#### Distribution method

The distribution method uses the imprecision value of the measurement in question (how reliable is the measurement) and results in the MCID being a value which is larger than this imprecision value, therefore being likely to represent a meaningful difference [10]. A common approach is to use test-retest data for an outcome [4]. This can help specify the size of the difference due to random variation in the measurement of the outcome.

#### Health economic method

This method tries to consider not only the MCID, but also the cost of the treatment and any other factors which are deemed to be important when deciding whether to run a trial. This method aims to establish a threshold value which is deemed acceptable for the cost per unit increase in health [11]. It estimates the relative efficiency of the treatments which can then be compared directly. This method is not commonly used in practice, with all 13 papers which used this method to establish the MCID using hypothetical datasets [4].

#### Opinion-seeking

This method is more intuitive, based on determining a value or a range of values for the clinically meaningful difference. This is established by asking clinicians or experts in the relevant fields to provide a professional opinion [4]. These experts could be patients [12, 13], clinicians or a combination [14], for example, with each providing a different perspective of what they deem important.

#### Pilot study

A pilot study is a small version of the trial which is being planned [15, 16]. Conventionally used to assess the feasibility of the main trial, though information can be collected to aid sample size calculation such as the effect size and population standard deviation [17, 18]. The effect size observed in a pilot study can be used as a starting point to help determine the MCID [4]. This method is commonly used but not often reported [4].

#### Review of evidence base

This method collects all existing evidence about the treatment area or population. This allows researchers to choose an important or realistic difference based on previous trials and research [19]. The optimum method used to do this is meta-analysis [4]; however, trialists should be wary of possible publication bias.

#### Standardised effect size

The standardised effect size is scale-invariant, which means that it can be generalised across a variety of clinical areas, it has no units of measurement [4]. For continuous outcomes, this is calculated by taking the difference in means and dividing by the pooled standard deviation [20]. Consider the difference between the two groups be *d*, and the pooled population standard deviation be *σ*, the standardised effect size (*δ*) can be calculated as:

$$ \delta =\frac{d}{\sigma }. $$

The size of the standardised effect is used to establish whether an important difference has occurred, which is conventionally 0.2 for a small effect, 0.5 for a moderate effect and 0.8 for a large effect [20]. The benefits of this method are that it is simple to calculate and allows for comparisons across different outcomes, trials, populations and disease areas [4].

These categories were taken from published work and allowed this study to complement the DELTA2 study currently being undertaken [21]. This work is being included in the DELTA2 study, hence the rationale for using the same categories for target difference elicitation.

### Calculating the standardised effect size

For a study with a continuous endpoint that follows a normal distribution, the standardised effect size is given by:

$$ \delta =\frac{d}{\sigma }, $$

where *δ* is the standardised effect size, *σ* is the standard deviation and *d* is the target difference.

For a conventional sample size calculation [22] for a given target sample size, power and significance level then the standardised target effect size can be calculated from:

$$ \kern0.75em \boldsymbol{\delta} =\frac{\sqrt{\mathbf{2}}\left({\boldsymbol{Z}}_{\mathbf{1}-\boldsymbol{\beta}}+{\boldsymbol{Z}}_{\mathbf{1}-\raisebox{1ex}{$\boldsymbol{\alpha} $}\!\left/ \!\raisebox{-1ex}{$\mathbf{2}$}\right.}\right)}{\sqrt{\boldsymbol{n}}}. $$

This calculation was used to calculate a scale-independent value for the target effect size for each study regardless of the clinical outcome.

The observed effect sizes were standardised using two methods to ensure similarity. Both these methods use the standard normal distribution properties of *p* values and test statistics.

The first method was based on the provided *p* value in the report. To calculate the standardised observed effect size, the following result was used:

$$ {d}_{observed}={\varPhi}^{-1}\left(p- value\right)\times \sqrt{\frac{1}{n_A}+\frac{1}{n_B}}. $$

Where *n*_*A*_ and *n*_*B*_ are the target sample size in each arm of the trial.

The second method depended on the type of primary outcome reported; however, this expanded on the first method. These calculations are given in Table 1.

**Table 1:** Calculations used on the extracted data to estimate the standardised observed effect size

Observed effect size type	Z-statistic calculation	Re-arrangement to get standardised observed effect size
Mean difference, Difference in proportions, Regression coefficient, Absolute risk reduction, Analysis of variance/covariance (ANOVA/ANCOVA) coefficients	$Z=\frac{d}{SE(d)}$	${d}_{observed}=Z\times \sqrt{\frac{1}{n_A}+\frac{1}{n_B}}$
Odds ratio	$Z=\frac{\ln \left[ OR\right]}{SE\left(\ln \left[ OR\right]\right)}$	${d}_{observed}=Z\times \sqrt{\frac{1}{n_A}+\frac{1}{n_B}}$
Risk ratio	$Z=\frac{\ln \left[ RR\right]}{SE\left(\ln \left[ RR\right]\right)}$	${d}_{observed}=Z\times \sqrt{\frac{1}{n_A}+\frac{1}{n_B}}$
Hazard ratio	$Z=\frac{\ln \left[ HR\right]}{SE\left(\ln \left[ HR\right]\right)}$	${d}_{observed}=Z\times \sqrt{\frac{1}{n_A}+\frac{1}{n_B}}$

### Statistical analysis

Summary statistics and graphs were used to describe the data. Expected and observed effect sizes were estimated using data extracted as discussed in the previous section. Statistical analyses were conducted using Microsoft Excel, R and IBM SPSS Version 23.

## Results

**Fig. 1:**
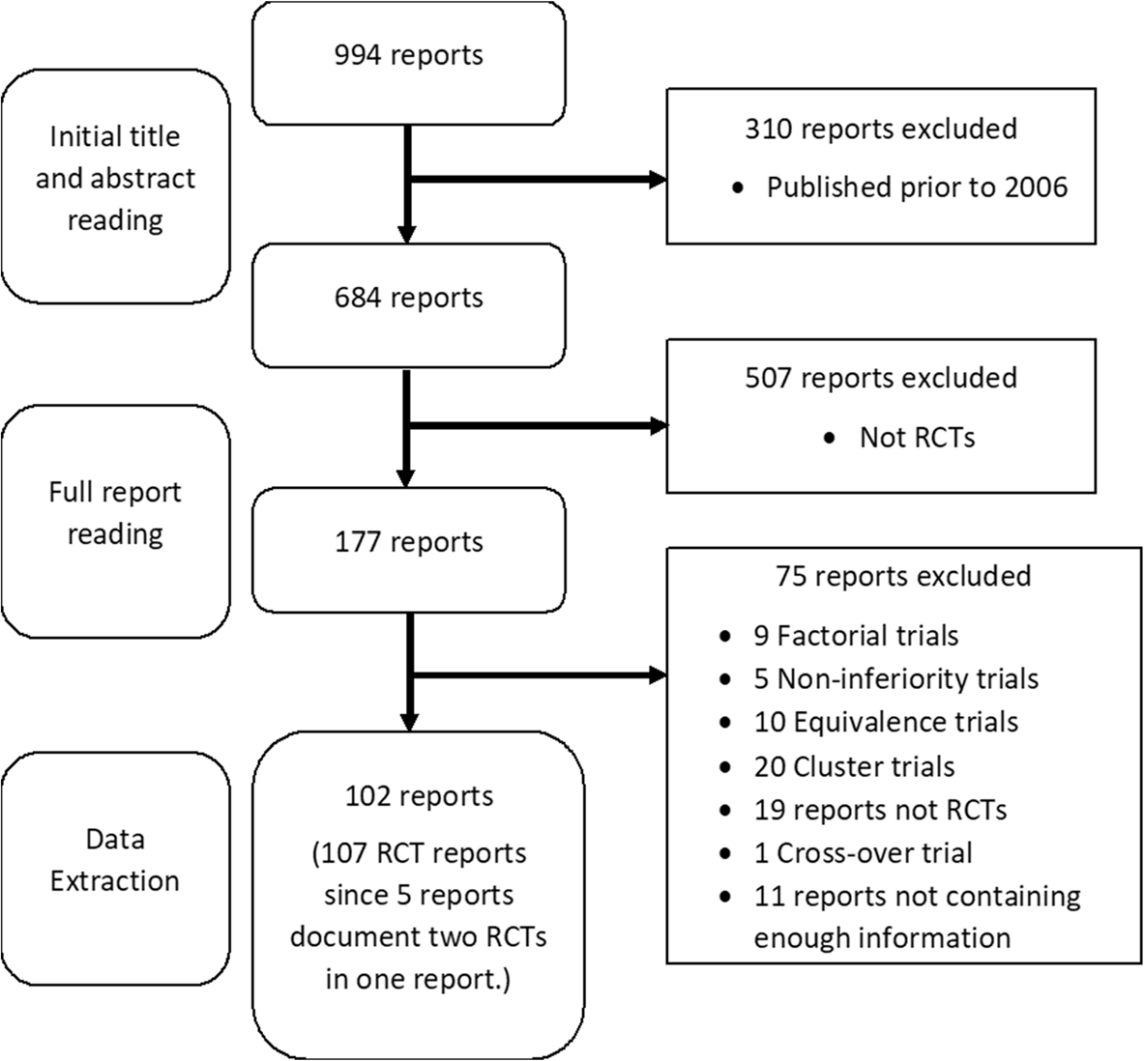
A flow chart displaying the inclusion of trials in the studyᅟ

The database contained information on 107 RCTs from 102 HTA reports. Trials were generally well-reported, with more information included in trials published after 2010 and after publication of the amended Consolidated Standards of Reporting Trials (CONSORT) Statement. Figure 1 gives the flow of trials through the various stages of the study.

### Trial characteristics

Table 2 summarises the characteristics of the included trials. It can be observed that the number of included trials increases with more recent volumes. Mental health was the most common clinical area (*N* = 18, 14.2%). A total of 35/107 (32.7%) studies reported statistically significant findings for the primary outcome measure.

**Table 2:** Summary characteristics of included trials

Characteristic	*N* (% of total RTCs)
Volume	
20 (2016)	20 (18.7)
19 (2015)	19 (17.8)
18 (2014)	12 (11.2)
17 (2013)	11 (10.3)
16 (2012)	8 (7.5)
15 (2011)	6 (5.6)
14 (2010)	8 (7.5)
13 (2009)	10 (9.3)
12 (2008)	2 (1.9)
11 (2007)	3 (2.8)
10 (2006)	8 (7.5)
Clinical area	
Cardiovascular	11 (10.3)
Critical Care	2 (1.9)
Dermatology	9 (8.4)
Diabetes	3 (2.8)
Gastrointestinal	9 (8.4)
Geriatrics	2 (1.9)
Immunology	2 (1.9)
Lifestyle	5 (4.7)
Mental Health	18 (14.2%)
Neurology	4 (3.7)
Obstetrics and Gynaecology	2 (1.9)
Oncology	4 (3.7)
Orthopaedics	6 (5.6)
Other	3 (2.8)
Paediatrics	9 (8.4)
Renal/Urology	6 (5.6)
Respiratory	7 (6.5)
Stroke	5 (4.7)
Reached statistical significance?	
Yes (*p* < 0.05)	35/107 (32.7%)
No	72/107 (67.3%)
Final target sample size	
Mean	1122
Median	432
Achieved sample size	
Mean	1015
Median	404

### Elicitation methods

The most commonly reported method of elicitation of the target effect size is the review of evidence method, as seen in Table 3. This was reported in 52.3% of reports (*N* = 56), either as the sole method or in combination with other methods. This elicitation method was the most common (or equal most common) in all clinical areas. However, in 19.6% of the reports there was no mention of the elicitation method used (*N* = 21).

**Table 3:** Summary statistics for elicitation method

DELTA elicitation method	Frequency	%
Anchor	0	0
Distribution	2	1.9
Health economics	1	0.9
Opinion-seeking	10	9.3
Pilot	4	3.7
Review of evidence	49	45.8
Standard effect size (SES)	5	4.7
Mixed^a^	7	6.5
No mention	21	19.6
Other	8	7.5

^a^‘Mixed’ methods included review of evidence

### Standardised effect sizes

Table 4 gives the average target and observed effect sizes after standardisation, overall and by statistical significance. This shows that the median standardised target effect size was 0.300 (IQR 0.198, 0.377). According to the standard categories of Cohen [20], (a small effect is 0.2, a moderate effect is 0.5 and a large effect size is 0.8), this corresponds to a small effect size. The largest standardised target effect size was 0.760; however, there were only two trials (1.9%) which used values above 0.600. The median standardised observed effect size is 0.112 (IQR 0.048, 0.287). The results when split by statistical significance behave as one would expect. The statistically significant median for observed effect size is larger than the target, whilst for the non-significant results it is considerably smaller.

**Table 4:** Standardised effect sizes of trials

Effect size	Median	(25th, 75th percentiles)	Minimum	Maximum
Overall				
Standardised target	0.300	0.198, 0.377	0.051	0.760
Standardised observed	0.112	0.048, 0.287	< 0.001	1.184
*p* < 0.05				
Standardised target	0.309	0.229, 0.433	0.051	0.643
Standardised observed	0.343	0.230, 0.501	< 0.001	1.184
*p* > 0.05				
Standardised target	0.297	0.183, 0.362	0.070	0.760
Standardised observed	0.061	0.019, 0.155	< 0.001	0.716

**Fig. 2:**
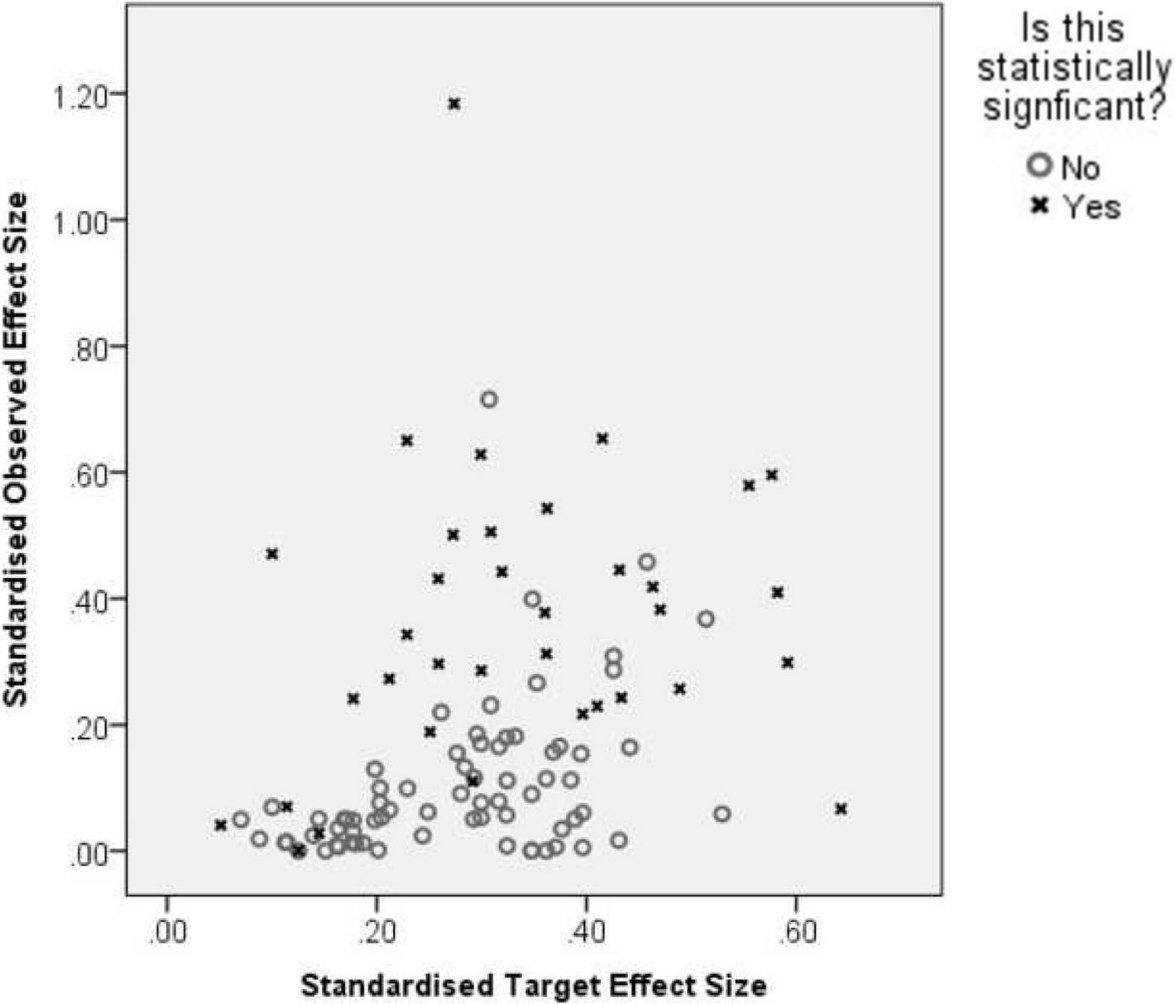
The standardised target and observed effect sizes in the trials, by statistical significance

Figure 2 gives the target and observed standardised effect sizes by whether the study reached statistical significance. This figure shows that the majority of trials which were not statistically significant had target effect sizes greater than the observed. This is what one would expect.

Table 5 gives the standardised expected and observed effect sizes by the type of primary endpoint used in the sample size calculation. It can be seen in Table 5 that a continuous endpoint is the most common type of primary endpoint (*N* = 49, 45.6%), closely followed by an endpoint on proportional scale (*N* = 41, 38.3%). Trials using continuous endpoints have higher average standardised observed effect sizes, as well as higher standardised target standardised effect sizes. There are three trials categorised as ‘Other’, two of which were mean area under the curve (AUC) across all patients, and one was an ordinal endpoint. The AUC trials were both across multiple time points, then the average AUC was taken as the primary endpoint, with one being a depression trial and the other being an ulcerative colitis trial.

**Table 5:** Standardised effect sizes by type of primary endpoint measure

Primary endpoint measure	Count	Standardised target effect size		Standardised observed effect size	
		Mean	Median	Mean	Median
Overall					
Continuous	49	0.375	0.353	0.277	0.219
Proportion	41	0.224	0.198	0.115	0.048
Time to event	10	0.291	0.312	0.147	0.065
Count	4	0.250	0.245	0.045	0.048
Other	3	0.295	0.295	0.169	0.186
*p* < 0.05					
Continuous	22	0.403	0.406	0.420	0.396
Proportion	11	0.234	0.258	0.285	0.312
Time to event	1	0.212	0.212	0.273	0.273
Count	1	0.114	0.114	0.070	0.070
Other	0				
*p* > 0.05					
Continuous	27	0.352	0.347	0.156	0.156
Proportion	30	0.220	0.192	0.052	0.027
Time to event	9	0.300	0.316	0.133	0.051
Count	3	0.296	0.377	0.036	0.035
Other	3	0.295	0.295	0.169	0.186

**Fig. 3:**
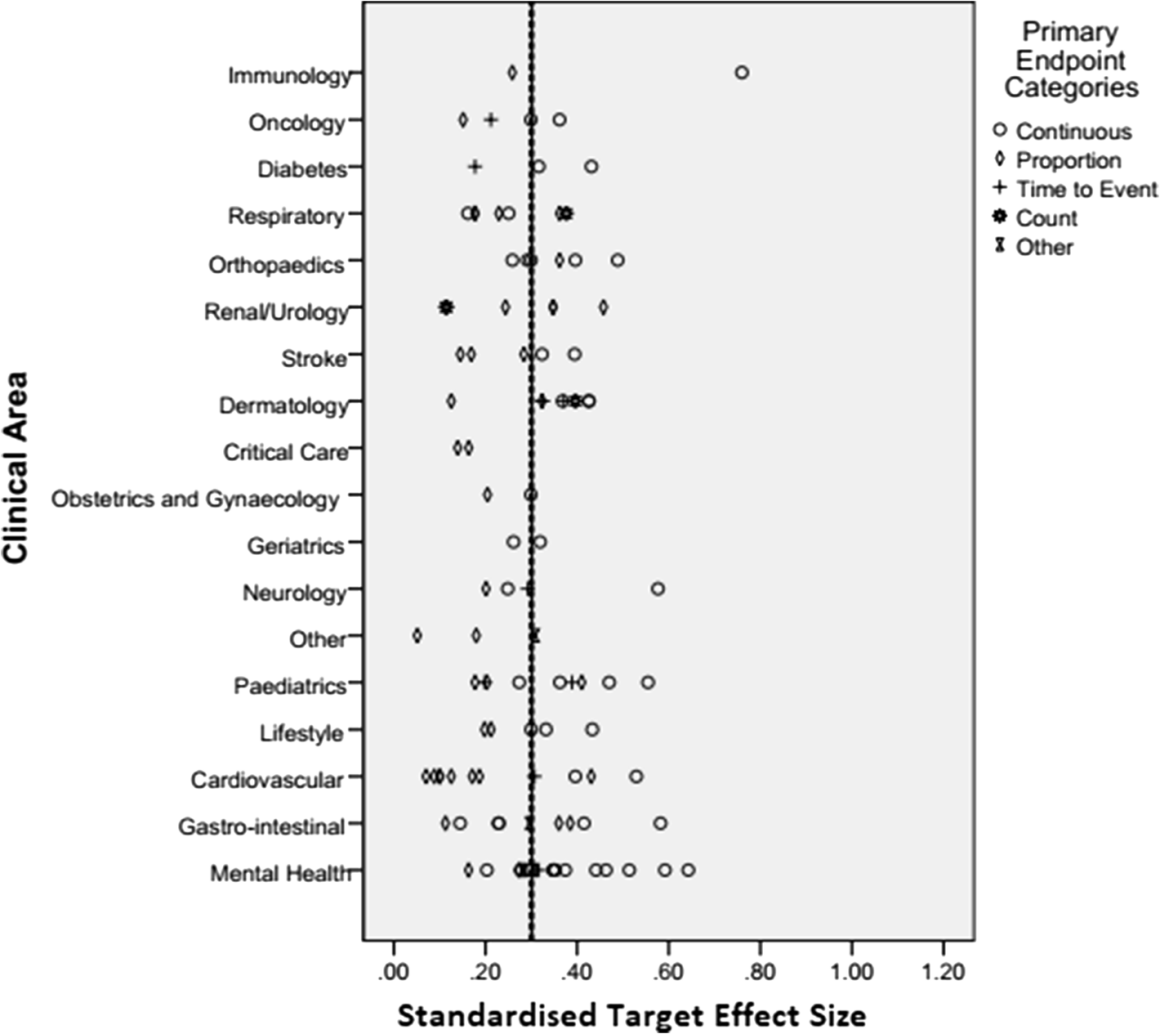
The standardised target effect size by clinical area and primary end-point

Figure 3 gives the observed standardised effect sizes for each clinical area including both the median and mean. Whilst the median effect size is relatively small, there are some extreme values. The separation of the mean and median lines indicate a skew in the data.

**Fig. 4:**
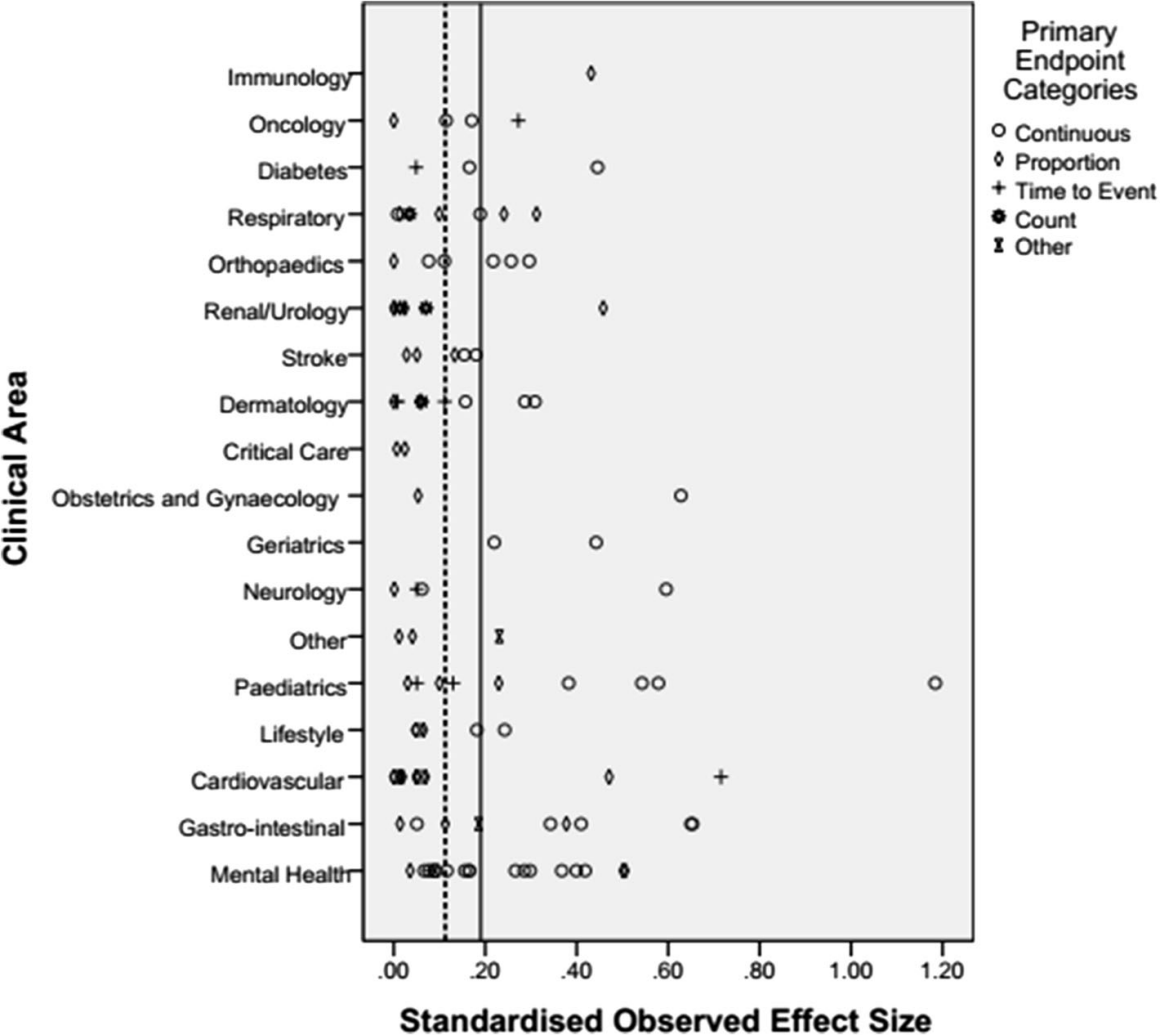
The standardised observed effect size by clinical area and primary end-point

Figure 4 gives the target standardised effect sizes for each clinical area. Both the mean and median are around 0.3, which corresponds to a small effect size in Cohen’s categories [8].

Table 6 gives the standardised target and observed effect sizes by clinical area. It can be noted that there is variation between the size of the effect sizes and clinical area, with areas such as cardiovascular and critical care using smaller target effect sizes than mental health, for example. It can be observed that, on average, investigators are anticipating effect sizes of between 0.2 and 0.4 for most clinical areas.

**Table 6:** Standardised target and observed effect sizes by clinical area

	Frequency	Standardised target effect size	Standardised observed effect size
	Count	Median	Median
Clinical area			
Cardiovascular	11	0.171	0.050
Critical care	2	0.151	0.016
Dermatology	9	0.368	0.061
Diabetes	3	0.316	0.166
Gastrointestinal	9	0.295	0.343
Geriatrics	2	0.290	0.331
Immunology	2	0.509	0.432
Lifestyle	5	0.300	0.065
Mental Health	18	0.332	0.165
Neurology	4	0.270	0.056
Obstetrics and Gynaecology	2	0.252	0.341
Oncology	4	0.255	0.143
Orthopaedics	6	0.331	0.164
Other	3	0.180	0.041
Paediatrics	9	0.362	0.230
Renal/Urology	6	0.296	0.019
Respiratory	7	0.229	0.009
Stroke	5	0.285	0.133

## Examples of good practice

A number of reports showed clearly the methods used to elicit the target effect size and are worthy examples of good practice. Two examples of good practice have been included to illustrate how the methods for quantifying the target difference can be described. They provide clear and transparent explanations of the journey to elicit the target effect size for their studies. They also utilised a variety of methods, including review of evidence and expert opinion, which have been recommended in the DELTA2 guidance for eliciting a realistic and important difference [23].

### TITRe2 trial

The TITRe2 trial (ISRCTN70923932) by Reeves et al. [24] gives the complex journey that eliciting the target effect size can be. The trialists’ used a variety of methods to estimate the target difference and clearly reports them all for the reader, as well as accounting for the uncertainty in the final estimate. An extract of the sample size calculation is given below.

The trial was designed to answer superiority questions. The following steps were taken to calculate the sample size.

From observational data, we assumed that approximately 65% of patients would breach the threshold of 9 g/dl and 20% would breach the 7.5 g/dl threshold. Therefore, with complete adherence to the transfusion protocol, we assumed that transfusion rates should be 100% in the liberal group and ≈ 30% (0.20/0.65) in the restrictive group.

In the observational analysis, 63% of patients with a nadir haematocrit between 22.5 and 27%, and 93% of patients with a nadir haematocrit below 22.5% were transfused. Therefore, in combination with the proportions of patients expected to breach the liberal and restrictive thresholds, these figures were used to estimate conservative transfusion rates of 74% for the liberal group and ≤ 35% for the restrictive group. These percentages reflected the rates of transfusion documented in the observational study (Fig. 1) and assumed non-adherence with the transfusion protocol of approximately 26% in the liberal group and 5% in the restrictive group.

The observational frequencies of infectious and ischaemic events for transfused and non-transfused patients were adjusted to reflect the estimated transfusion rates in the two groups (i.e. 74 and ≤ 35%), giving event rates for the proposed composite outcome of 17% in the liberal threshold group and 11% in the restrictive threshold group. A sample size of 1468 was required to detect this risk difference of 6% with 90% power and 5% significance (two-sided test), using a sample size estimate for a chi-squared test comparing two independent proportions (applying a normal approximation correction for continuity) in Stata version 9.

The target sample size was inflated to 2000 participants (i.e. 1000 in each group) to allow for uncertainty about non-adherence and the estimated proportions of participants experiencing the primary outcome. We regarded these parameter estimates as uncertain because (1) they were estimated from observational data, (2) they were based on the red blood cell transfusion rate only in Bristol, (3) they were based on routinely collected data, using definitions for elements of the composite primary outcome which are not identical to those proposed for the trial and (4) they were based on any compared with no red blood cell transfusion, rather than on the number of units of red blood cells likely to be transfused in participants who breach the liberal threshold. No adjustment was made for withdrawals or loss to follow-up, as both rates were expected to be very low.

We expected approximately two thirds of participants to breach the haemoglobin threshold for eligibility. Therefore, we predicted that we needed to register approximately 3000 participants into the study as a whole to allow 2000 participants to be randomised into the main study.

The main outcome measure for the economic evaluation was quality-adjusted life years (QALYs), which are derived from EQ-5D-3L utilities measured on a continuous scale and time under observation. The analysis of QALYs required baseline utility to be modelled as a covariate; the correlation between baseline and 3-month EQ-5D-3L utilities was assumed to be ≥ 0.3 With a total sample size of 2000, the trial had more than 95% power to detect a standardised difference in continuous outcomes between groups of 0.2 with 1% significance (two-sided test). This magnitude of difference is conventionally considered to be ‘small’.

Following personal correspondence with the chief investigator (B Reeves), it was clarified that the process was done prospectively. The team spent a lot of time when designing the trial before reaching the decision to consent the patients before the surgery and randomise after surgery; this decision facilitated recruitment but made randomisation 24/7 challenging to implement and resulted in over 40% of consented patients being ineligible for randomisation (i.e. did not breach the liberal threshold). Professor Reeves highlighted how from his experience, ‘target difference’ is an alien concept to many clinicians which results in him regularly reverting to a ‘bracketing’ method, which is a standard method in psychophysics for estimating a threshold, to hone in on a target threshold difference which a clinician believes to be important. This discussion highlights the importance of communication within a study team and the challenges regularly encountered when trying to elicit a target effect size for a sample size calculation.

### CADET trial

One trial which reported using a pilot study to aid the elicitation of the target effect size was by Richards et al. [25], the CADET trial (ISRCTN32829227). This study was a cluster trial; therefore, it was excluded from the full study. However, initially cluster trials were being included since they are an extension of individual RCTs so data extraction was completed on this report. The trial was investigating the effectiveness of collaborative care for depression in primary care.

We powered the trial at 90% (alpha = 0.05) to detect an effect size of 0.4, which we regarded as a clinically meaningful difference between interventions. This figure was within the 95% confidence interval (CI) of the effect predicted from data collected during our pilot work (effect size 0.63, 95% CI 0.18 to 1.07). To detect this difference would have required 132 participants per group in a two-armed participant-randomised trial.

For our cluster trial, with 12 participants per primary care cluster and an intra-cluster correlation (ICC) of 0.06 from our pilot trial, the design effect was 1.65 leading to a sample size of 440. To follow up 440 participants, we aimed to randomised 550 participants (anticipating 20% attrition).

The trial observed an effect size of 0.26 but reached statistical significance (*p* = 0.009). The ‘Discussion’ section in the paper details that whilst the observed effect size was less than the one which the study was powered on the 95% CI around the observed effect size included the target effect size. It also discussed that the observed effect size was also within the CI of the smallest meaningful difference in a recent meta-analysis.

After further discussion with the trial statistician, it was clarified that the trial was designed based on a clinically meaningful effect size of 0.4, which was independently identified. This was shown in the trial protocol [26], which referenced two trials, a review and a clinical opinion to estimate the target effect size. The pilot study was used to demonstrate that a UK version of collaborative care might be likely to achieve such an effect, in line with collaborative care interventions in other countries such as the USA.

This use of multiple methods to estimate the target effect size shows how thorough review of previous work as well as an understanding of each of the methods can benefit the estimation of the target difference.

## Discussion

The study in this paper gives an indication of the most commonly reported methods for target difference elicitation as well as the use of multiple methods. This study demonstrates what trialists’ are reporting and the journey they take to establish the target effect size.

We found that the most commonly used method was the review of evidence method, so using previously published research to aid the quantification of the anticipated effect size. This method was also used in tandem with other methods, resulting in an overall percentage of use of 52.3%.

The average standardised target effect sizes in the trials was 0.300, which corresponds to a small effect. Only five studies had a target effect size greater than 0.600. The average observed effect size was 0.112, with the largest observed effect being 1.200 and only two studies observing effect sizes greater than 0.600. These results should be used when reviewing grant applications and trials to determine if the target difference specified is realistic.

The difference between the observed and anticipated effect sizes is as expected since half of all studies are not statistically significant [27]. In this study, 67.3% of studies gave a non-significant result. The observed effect was larger than the target effect size in 19.6% of trials. A relatively high proportion of published HTA-funded studies are meeting their target effect size, though the effect sizes were small in all clinical areas.

Based on the case studies, it is clear that transparency is required when discussing an estimated target effect size. It could be that some trialists do not want to report that they used multiple methods, whereas the use of multiple methods of elicitation should result in a more accurate estimate.

There were 19.6% of reports which did not discuss where their target effect size came from. Since previous research is used so frequently in target effect size elicitation, and with other published research not stating where the target effect size came from, this could result in future trials using previous research which has no founding or reason for the chosen effect size, which is a cause for concern.

With the TITRe2 trial, the slight inflation of the sample size to account for the uncertainty of the observational data seems to be a sensible approach and is to be recommended.

One limitation of this study is that the trials are all UK based. However, this should not affect the generalisability of the results. Even though only one journal was used in this study, this particular journal captures high-quality trials in the UK and thus the results are generalisable. A potential implication of the high-quality of reporting is that a larger amount of information is captured compared to other journals. Whilst this could be deemed a limitation of generalizability of results, these results paint a clear picture of what is occurring currently in clinical trials.

## Conclusion

This study provides evidence that the median target effect size is 0.300 in publicly funded-HTA trials in the UK. It is recommended that there should be transparency in the quantification of the target effect size in clinical trials and that the results in this paper on the median effect sizes should be used to assess if a stated effect size is realistic.

## Appendix

Study ID

Study acronym (if provided)

Full study title

Lead author

Corresponding author

Publication year, volume, issue

ISRCTN

Trial type and design

Is the trial randomised?

Is the trial multicentre?

What clinical area is the trial investigating?

Number of arms

Trial population

Setting

- Hospital
- GP
- Mixed
- Community
- Primary or secondary care
- Other

Primary endpoint and measure type

Intervention type

\begin{itemize}

- Drug
- Therapy
- Surgical
- Education
- Complex
- Other

Control type

- Missing
- Active
- Placebo
- Not applicable

Target (unadjusted) and final target sample size

Achieved and evaluable sample size

Target power and significance level

Expected difference and standard deviation (if provided)

Expected effect size

Elicitation method

- Previous research
- Pilot
- Systematic review
- Cochrane review
- Expert consensus
- Meta-analysis
- Other
- No mention

Is the expected effect size the minimally important clinical difference (MCID)?

If previous study used to elicit target, what was result observed in study?

DELTA categories of elicitation

- Anchor
- Distribution
- Health economics
- Opinion-seeking
- Pilot
- Review of evidence
- SES (standardised effect size) (Cohen)
- Mixed
- No mention
- Other

Observed treatment effects in each arm

Observed effect size

Observed effect size type

- Mean difference
- Relative risk
- Odds ratio
- Hazard ratio
- Difference in proportions
- Regression coefficient
- Difference in score
- General Linear Model coefficient
- Other

*P* value

Is the *p* value significant?

Lower and upper 95% confidence interval boundaries

There were sections provided for free text about the following areas

- Trial design
- Early comments from initial reading
- Trial population
- Clinical area
- Intervention type
- Primary endpoint
- Sample size
- Target difference elicitation
- Treatment difference
- Observed effect size
- If the *p* value was non-significant for a MCID, was MCID re-evaluated?
- Further comments

## Abbreviations

AUC: Area under the curve
CI: Confidence interval
ENT: Ear, nose and throat
HTA: Health Technology Assessment
IQR: Interquartile range
MCID: Minimum clinically important difference
MRC: Medical Research Council
NIHR: National Institute of Health Research
QALY: Quality-adjusted life year
QoL: Quality of life
RCT: Randomised controlled trial
SES: Standardised effect size
UK: United Kingdom
